# Association of Infliximab and Vedolizumab Trough Levels with Reported Rates of Adverse Events: A Cross-Sectional Study

**DOI:** 10.3390/jcm10184265

**Published:** 2021-09-20

**Authors:** Ido Veisman, Oranit Barzilay, Liora Bruckmayer, Ola Haj-Natour, Uri Kopylov, Rami Eliakim, Shomron Ben-Horin, Bella Ungar

**Affiliations:** 1 Sheba Medical Center Tel Hashomer, Department of Gastroenterology, Ramat Gan 52620, Israel; idoweiss37@gmail.com (I.V.); oranitbarzilay@gmail.com (O.B.); liora.bruck@gmail.com (L.B.); ola.hajnatour@sheba.health.gov.il (O.H.-N.); ukopylov@gmail.com (U.K.); ramieliakim@yahoo.com (R.E.); shomron.benhorin@gmail.com (S.B.-H.); 2 Sackler School of Medicine, Tel-Aviv University, Tel Aviv-Yafo 67011, Israel

**Keywords:** IBD, infliximab, vedolizumab, biological therapy

## Abstract

Infliximab and vedolizumab are effective treatments for inflammatory bowel disease (IBD), although associated with adverse events (AE). While low or non-existent drug levels and positive antidrug antibodies have been associated with therapeutic failure, there is no clear association between higher drug levels and AE. A cross-sectional study consisting of Crohn’s disease (CD) and ulcerative colitis (UC) patients receiving infliximab or vedolizumab at the Sheba Medical Center was performed. Patients completed a questionnaire regarding AEs related to biological therapy. Serum trough levels obtained on the same day were analyzed. Objective measures of outcomes were retrieved from medical records. Questionnaires were completed by infliximab (n = 169) and vedolizumab (n = 88)-treated therapy patients. Higher infliximab levels were only numerically associated with the occurrence of at least one AE (*p* = 0.08). When excluding fatigue and abdominal pain, higher infliximab levels were statistically associated with the occurrence of at least one AE (*p* = 0.03). Vedolizumab drug levels > 18 μg/mL were also linked with the occurrence of more AEs. No specific association was observed between the increased levels of either infliximab or vedolizumab and specific AEs (neurological symptoms, upper GI symptoms, infectious complications, and musculoskeletal symptoms). As significant AEs are very rare, additional multi-center studies are required.

## 1. Introduction

Over the last two decades, biological therapies have revolutionized treatment paradigms in various chronic inflammatory diseases, including IBD. Commonly used biologic agents proven effective in IBD treatment are monoclonal antibodies to tumor necrosis factor alpha (TNF) and anti-integrin agents. While these treatments have been shown to be highly effective in IBD, they are not risk-free. Anti-TNF agents are associated with a wide range of adverse events (AEs), the main ones being the increased risk of infections and potential risk of lymphoma [1]. Vedolizumab is a selective anti-α4β7 integrin agent that affects specifically the gastrointestinal (GI) tract. However, the effects on other systems cannot be ruled out [2]. Clinical trials have shown higher rates of nasopharyngitis among Crohn’s disease (CD) patients receiving vedolizumab (GEMINI II) [3,4]. Nevertheless, in a comprehensive study covering over 200,000 patient years of vedolizumab exposure, based on the Vedolizumab Global Safety Database, 13% of AEs were defined as serious, the main ones being clostridium difficile infection and pneumonia [5].

The therapeutic monitoring of anti-TNF therapy has been well established in UC and CD [6], and guides therapeutic decisions regarding dose optimization, discontinuation of biological treatment, or the need for combination therapy with immunomodulators [7]. Although low or non-existent drug levels and positive antidrug antibodies have been associated with therapeutic failure and less favorable outcomes, there is no clear consensus as to the possible associations between higher drug levels and AE. In a previous retrospective study by Greener and colleagues, higher infliximab serum concentrations were not associated with the increased risk for infections [8]. Regarding vedolizumab, although a correlation has been detected between the trough levels and clinical outcome [9,10], scarce data exists as to the possible effect of higher vedolizumab levels on adverse events. Therefore, the aim of the present study was to assess the association between infliximab and vedolizumab trough levels and the rate of various adverse events in CD and UC patients.

## 2. Materials and Methods

### 2.1. Study Design and Population

A cross-sectional study was performed. The study population consisted of CD and UC patients receiving regular infliximab or vedolizumab infusions at the gastroenterology institute of the Sheba Medical Center between 2019 and 2020.

Each participant completed a questionnaire regarding possible AEs. Serum trough levels were obtained concomitantly by filling out the questionnaire (Appendix A). The study examined the occurrence of adverse events versus concomitant trough levels; hence, patients who filled out the questionnaire more than once were included in the study. In total, 195 out of 476 patients treated with infliximab/vedolizumab at our center were randomly recruited upon their arrival at the infusion center.

Clinical and demographical data were obtained from each patient’s medical record. A retrospective examination of the patients’ medical files was performed 6 months after filling out the questionnaire to record significant AEs, leading to changes in the treatment regimen/hospitalization/antibiotic treatment.

#### 2.1.1. Study Outcomes

The study’s primary outcome was to examine infliximab/vedolizumab trough levels in association with possible AEs, as reported by patients.

AE occurrence was analyzed with regard to several aspects:

1. occurrence of any adverse events;
2. number of adverse events per patient; and
3. analysis per specific adverse event (Appendix A).

Secondary outcomes included the association of trough levels (TL) with adverse events, as retrieved from patients’ medical records. 

#### 2.1.2. Definitions

Adverse events were defined as experiencing one of the following manifestations following previous infliximab/vedolizumab infusion: rash/pruritus, dizziness, headache, paresthesia, nausea or vomiting, fever, pharyngitis, urinary tract infection, pneumonia, myalgia, bone pain, muscle weakness, abdominal pain, cough, dyspnea, sinusitis, blushing, increased fatigue, arthralgia, arthritis, and peripheral edema (Appendix A). Occurrence of at least one AE was defined as patients who reported one AE or more in the questionnaire.

Severe AEs were defined as those requiring medical intervention or cessation of biological therapy.

The inclusion criteria consisted of the following:

- established diagnosis of Crohn’s disease or ulcerative colitis (based on endoscopy and histology or imaging);
- regular infliximab or vedolizumab treatment at the Sheba Medical Center in light of the above diagnosis;
- patients under maintenance treatment who have completed induction protocol (weeks 0, 2, and 6);
- available clinical, demographic, and clinical data, as well as drug trough levels; and
- age > 18 years.

### 2.2. Measurement of Vedolizumab Concentrations

Vedolizumab concentration measurements were performed as previously described [11]. In short, integrin alpha4-beta7 (R&D systems, Minneapolis, MN, USA) was added to pre-plated anti-His-tag (R&D systems, Minneapolis, MN, USA) wells of ELISA plates (Nunc, Roskilde, Denmark). Serum was added and incubated. Plates were then washed and goat anti-human κ chain horseradish peroxidase (HRP)-labeled antibody (Serotec, Oxford, UK) was added. The results were read by an ELISA reader EL-800 (Biotek Instruments, Winooski, USA) and expressed as μg/mL.

### 2.3. Measurement of Infliximab Concentrations

Infliximab concentration measurements were performed as previously described [12]. In short, serum was added to pre-plated TNFα wells (Peprotech, Rocky Hill, NJ, USA) and incubated. Following washing, HRP-labeled goat anti-human Fc fragment antibody (MP Biomedicals, Solon, OH, USA) was added and reacted with the tetramethylbenzidine (TMB) substrate. The results were then read on an ELISA reader. 

### 2.4. Statistical Analysis

Continuous variables were expressed as the median and interquartile range (IQR), and categorical variables as a percentage. The Mann–Whitney test was used to compare continuous variables and Fischer’s exact test was used for categorical data. All reported *p*-values were two-sided and a *p*-value of less than 0.05 was considered statistically significant. All statistical calculations were performed with the use of MedCalc software (version 12.2.1.0, Mariakerke, Belgium).

### 2.5. Ethical Considerations

The study was approved by the Sheba Medical Center ethics committee. All patients signed an informed consent form for the sera analyses and review of medical records.

## 3. Results

### 3.1. Patient Population and Demographics

Between March 2019 and January 2020, 372 IBD patients were treated with infliximab and 104 were treated with vedolizumab at the Gastroenterology Institute at the Sheba Medical Center. Questionnaires assessing adverse events were completed by infliximab and vedolizumab-treated therapy patients. Among the 169 questionnaires filled by infliximab-treated patients, 84 patients completed the questionnaire once, 35 patients completed the questionnaire twice, and five patients completed the questionnaire three times (i.e., 124 infliximab-treated IBD patients participated in this study). Regarding the 88 questionnaires completed by vedolizumab-treated patients, 56 completed the questionnaire once, 13 completed the questionnaire twice, and two patients completed the questionnaire three times (i.e., 71 vedolizumab-treated IBD patients participated in this study). All demographic and clinical data of infliximab and vedolizumab-treated patients are depicted in Table 1.

### 3.2. Association of Drug Trough Levels with Occurrence of Adverse Events

#### 3.2.1. Infliximab

**Figure 1:**
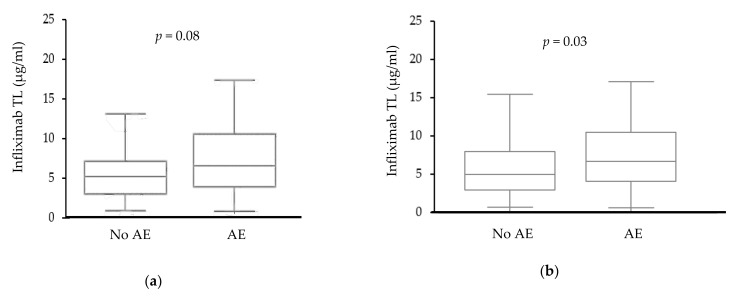

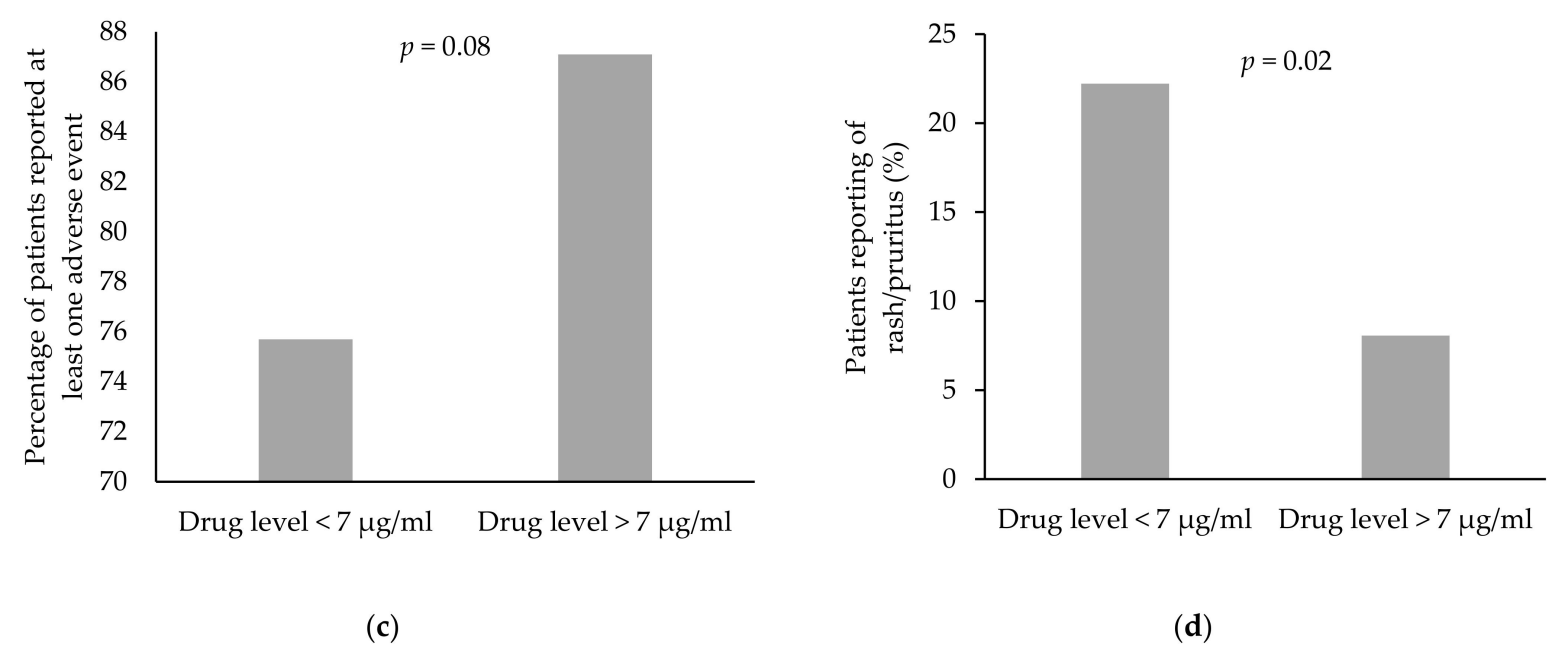
Association between infliximab serum drug levels and the reporting of adverse events (AEs). (**a**) Association between infliximab levels and reporting of at least one AE. (**b**) Association between infliximab levels and reporting of at least one AE, excluding fatigue and abdominal pain. (**c**) Association between infliximab trough levels of below or above 7 µg/mL and reporting of at least one AE. (**d**) Percentage of patients reporting of a new rash or pruritus among patients with infliximab trough drug levels below or above 7 µg/mL. Abbreviations: AE = adverse event; and TL = trough level.

The median of infliximab TL was 5.3 μg/mL (IQR 3.1–9.1 μg/mL) in the study cohort. Higher infliximab levels were numerically associated with the occurrence of at least one AE, although statistical significance was not reached (median trough levels among patients with at least one reported AE versus those not reporting an AE: 5.7 and 4.3 μg/mL, IQR 3.1–9.6 and 2.2–6.2 μg/mL, respectively, *p* = 0.08, Figure 1a). Nevertheless, when excluding fatigue and abdominal pain, higher infliximab levels were statistically associated with the occurrence of at least one AE (median trough levels among patients with at least one reported AE versus those not reporting an AE: 6 and 4.3 μg/mL, IQR 3.4–9.8 and 2.3–7.0 μg/mL, respectively, *p* = 0.03, Figure 1b). Six patients developed anti-infliximab antibodies (infliximab levels: median = 0.1 μg/mL, range = 0.04–5.7 μg/mL; antibodies levels: median = 4.3 μg/mL, range = 2.6–7.2 μg/mL), four (66%) of whom reported at least one AE. 

Specific adverse events were also studied in relation to trough levels. No significant differences in TL were observed in patients with and without rash or pruritus (median trough levels among patients who reported a new rash or pruritus versus those not who did not report a rash or pruritus: 6 and 5.3 μg/mL, IQR 1.9–9.8 and 3.1–8.8 μg/mL, respectively, *p* = 0.65); neurological symptoms (dizziness, headache, and paresthesia; median trough levels among patients who reported neurological symptoms versus those who did not report neurological symptoms: 6 and 4.8 μg/mL, IQR 3.0–9.8 and 3.0–8.4 μg/mL, respectively, *p* = 0.23); upper GI symptoms (nausea or vomiting; median trough levels among patients who reported upper GI symptoms versus those who did not report upper GI symptoms: 6 and 5.3 μg/mL, IQR 3.1–10.4 and 3.0–8.6 μg/mL, respectively, *p* = 0.25); infectious complications (including fever, pharyngitis, urinary tract infections, and pneumonia; median trough levels among patients who reported infectious complications versus those who did not report infectious complications: 4.7 and 5.4 μg/mL, IQR 1.4–6.1 and 3.3–9.2 μg/mL, respectively, *p* = 0.21); or musculoskeletal symptoms (including myalgia, bone pain, and muscle weakness; median trough levels among patients who reported musculoskeletal symptoms versus those who did not report musculoskeletal symptoms: 5.6 and 5.3 μg/mL, IQR 2.9–8.4 and 3.2–9.3 μg/mL, respectively, *p* = 0.76). 

As recent studies have associated infliximab TL of 7 μg/mL and above with optimal therapy outcome [13], a specific analysis focusing on this threshold was performed; drug levels above or below 7 μg/mL were not associated with the occurrence of at least one AE (OR 2.2, CI 0.9–5.1, *p* = 0.08, Figure 1c). Infliximab-treated patients with trough levels below 7 μg/mL reported a new rash or pruritus significantly more often than patients with infliximab levels above 7 μg/mL (29 patients reported a new rash or pruritus, of which 24 patients had infliximab levels below 7 μg/mL, OR 0.3, Cl 0.1–0.8, *p* = 0.02, Figure 1d). No associations were observed between neurological symptoms or upper GI symptoms and infliximab levels above or below 7 μg/mL (*n* = 31 versus 42 patients, *n* = 11 versus 12 patients; OR 1.5 and 1.725, Cl 0.8–2.9 and 0.7–4.1, *p* = 0.16 and *p* = 0.22, respectively). Furthermore, associations between infectious complications or musculoskeletal symptoms and infliximab trough levels were insignificant also (infectious complications: in seven and twelve patients with TL above versus below 7 μg/mL, OR 1.0, Cl 0.3–2.7, *p* = 0.97; musculoskeletal symptoms: in 19 and 23 patients with TL above versus below 7 μg/mL, OR 1.6, Cl 0.8–3.3, *p* = 0.17).

#### 3.2.2. Vedolizumab

The median of vedolizumab TL was 47.9 μg/mL (IQR 28.2–87.2 μg/mL) in the study cohort. As anti-vedolizumab antibodies are rare and inconclusive with regard to clinical outcome, they were not measured in the current study [11].

**Figure 2:**
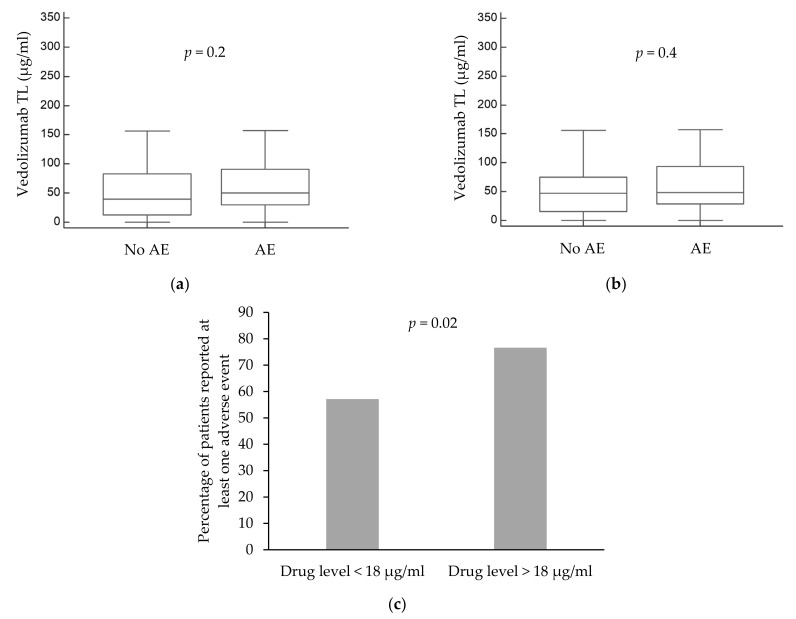
Association between vedolizumab serum drug levels and the reporting of adverse events (AEs). (**a**) Association between vedolizumab levels and reporting of at least one AE. (**b**) Association between vedolizumab levels and reporting of at least one AE, excluding fatigue and abdominal pain. (**c**) Association between vedolizumab trough levels of below or above 18 µg/mL and reporting of at least one AE. Abbreviations: AE = adverse event; and TL = trough level.

Among vedolizumab-treated patients, trough levels were not associated with the occurrence of at least one AE (median vedolizumab levels among patients with and without at least one reported AE: 50.4 and 40 μg/mL, IQR 29.6–90.4 and 12.4–83 μg/mL, respectively, *p* = 0.22, Figure 2a). When excluding fatigue and abdominal pain, vedolizumab drug levels were also not associated with the occurrence of at least one AE (median trough levels among patients with at least one reported AE versus those who did not report an AE: 47.6 and 47 μg/mL, IQR 29.1–92.3 and 15.7–73.5 μg/mL, respectively, *p* = 0.4, Figure 2b). 

No significant differences in TL were observed in patients with and without neurological symptoms (dizziness, headache, and paresthesia; median trough levels among patients who reported neurological symptoms versus those who did not report neurological symptoms: 60.5 and 44.3 μg/mL, IQR 36.4–102.5 and 22.8–83.7 μg/mL, respectively, *p* = 0.14); infectious complications (including fever, pharyngitis, urinary tract infections, and pneumonia; median trough levels among patients who reported infectious complications versus those who did not report infectious complications: 33 and 50.6 μg/mL, IQR 23.0–98.2 and 29.6–85.4 μg/mL, respectively, *p* = 0.35); or musculoskeletal symptoms (including myalgia, bone pain, and muscle weakness; median trough levels among patients who reported musculoskeletal symptoms versus those who did not report musculoskeletal symptoms: 50.4 and 47 μg/mL, IQR 37.4–85.2 and 22.5–95.7 μg/mL, respectively, *p* = 0.72).

Nevertheless, as vedolizumab levels above 18 μg/mL were previously reported to correlate with better therapy outcome [14,15], we explored drug levels above 18 μg/mL in association with AEs. More patients with trough levels above 18 μg/mL reported at least one AE compared to patients with levels lower than 18 μg/mL (OR 4.2, CI 1.2–14.8, *p* = 0.02, Figure 2c). 

Regarding specific adverse events, neither neurological symptoms (dizziness, headache, and paresthesia), upper GI symptoms (nausea or vomiting), nor dermatological manifestations (rash or pruritus) were associated with vedolizumab trough levels above or below 18 μg/mL (neurological symptoms: OR 3.6, CI 0.7–17.5, *p* = 0.11; upper GI symptoms: OR 1.2, CI 0.2–6.4, *p* = 0.76; and dermatological manifestation: OR 0.3, CI 0.05–2.08, *p* = 0.24). Similarly, vedolizumab levels were not associated with infectious complications or musculoskeletal symptoms (infectious complications: OR 0.8, CI 0.2–3.4, *p* = 0.82; musculoskeletal symptoms: OR 1.3, CI 0.3–4.7, *p* = 0.63).

ROC curves were performed for both drugs, assessing the occurrence of adverse events above drug threshold levels of 7 and 18 μg/mL for infliximab and vedolizumab, respectively. Findings were insignificant for both drugs (*p* = 0.69, AUC = 0.5 and *p* = 0.58, AUC = 0.5 for infliximab and vedolizumab, respectively).

### 3.3. Drug Trough Levels and Serious AEs

A sub-analysis was performed for the association of trough levels and adverse events. Significant clinical outcomes and adverse events were defined as those requiring medical intervention or cessation of biological therapy. The association of trough levels with several endpoints and the need for hospitalization, antibiotic treatment, steroid treatment, and change in biological treatment regimen was explored.

#### 3.3.1. Infliximab

In terms of adverse events, two patients (1%) presented with tonsillitis and pneumonia (TL 2.3 μg/mL and 8.6 μg/mL respectively), which required antibiotic therapy. 

In terms of clinical outcomes, among the patients treated with infliximab, four (2.5%) were hospitalized (TL range 5.7 μg/mL–21 μg/mL), while 16 patients (9.5%) underwent a change in treatment regimen due to active disease (median TL 3.3 μg/mL, IQR 0.2–9.1 μg/mL). In nine cases, dose escalation or interval shortening (median TL 3.3 μg/mL, IQR 3.3–5.6 μg/mL) was applied and seven were switched to another biological drug (median TL 3.6 μg/mL, IQR 0.1–11.2 μg/mL).

#### 3.3.2. Vedolizumab

In terms of adverse events, only two patients (2%) were treated with antibiotics: one due to Campylobacter infection and the second for an unspecified infectious exacerbation (TL 33.5 μg/mL and 3.3 μg/mL respectively). No other adverse events occurring during hospitalization, antibiotic treatment, or steroid treatment, or change in biologic treatment were reported.

In terms of clinical outcomes, three patients (3%) were hospitalized (TL range 33.5 μg/mL–130 μg/mL). Nine patients (11%) underwent a change in treatment regimen (median TL 50.4 μg/mL, IQR 33.5–130 μg/mL), which included drug replacement to a different biologic agent (7 patients), shortening the interval of drug administration (one patient) or systemic steroid administration (one patient).

### 3.4. Association of Patient-Specific Characteristics with the Occurrence of Adverse Events

**Figure 3:**
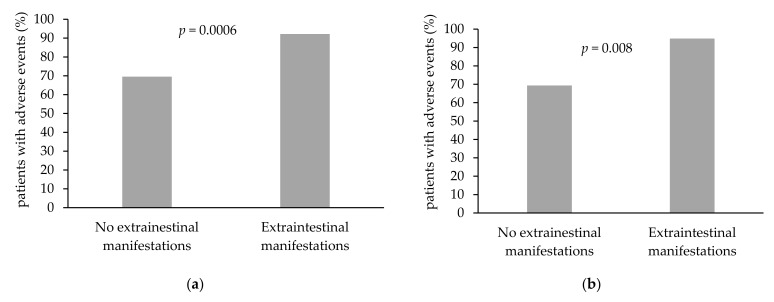

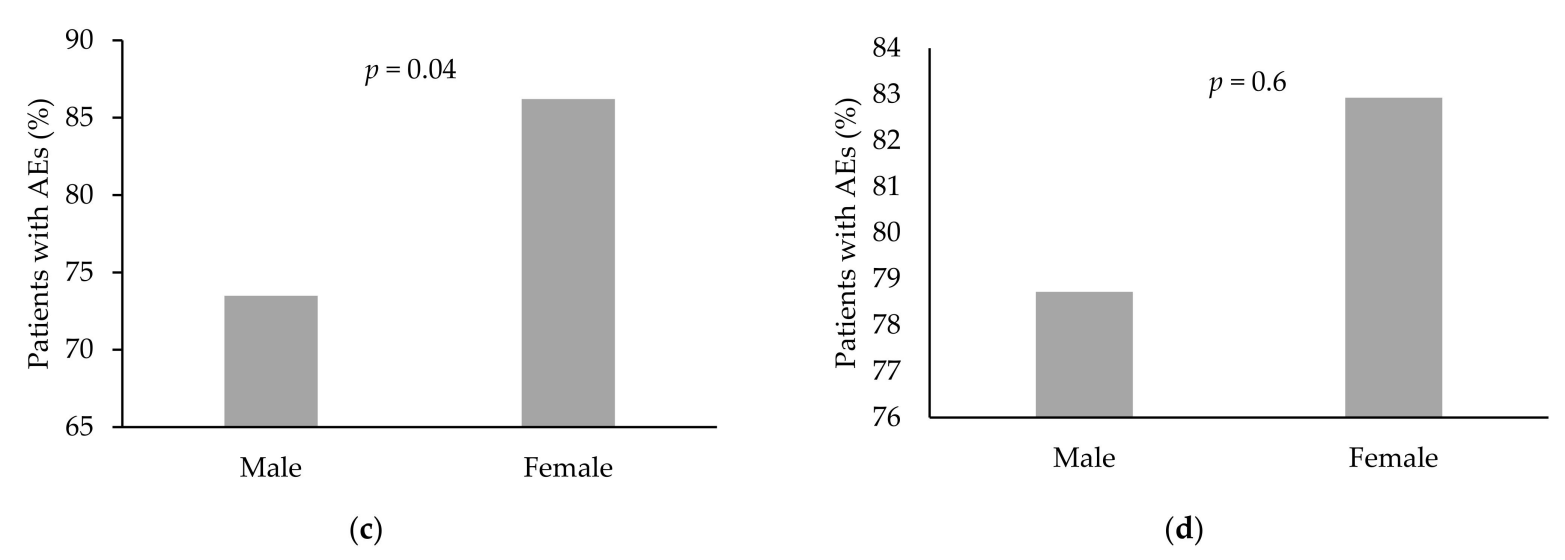
Association of extraintestinal manifestations and gender with the reporting of adverse events (AEs). (**a**) Percentage of AE among infliximab-treated patients with and without extraintestinal manifestations (*p* = 0.0006, OR 5.1, CI 2.0–13.3). (**b**) Rate of AE among vedolizumab-treated patients with and without extraintestinal manifestations (*p* = 0.008, OR 8.1, CI 1.7–38.3). (**c**) Percentage of AEs among male versus female patients treated with infliximab (*p* = 0.04, OR 2.2, CI 1.0–4.9). (**d**) Percentage of AEs among male versus female patients treated vedolizumab (*p* = 0.6, OR 1.3, CI 0.4–3.8). Abbreviation: AEs = adverse events.

Patients with known extraintestinal manifestations (EIM) demonstrated more AEs, (71/77, 92.2% for infliximab; 37/39, 94.9% for vedolizumab) compared to patients without known EIM (*p* = 0.0006 and *p* = 0.008, as in Table 2, Figure 3a,b, for infliximab and vedolizumab, respectively). Parameters such as age, disease duration, IBD type, disease location, disease phenotype, previous surgery, concomitant immunomodulatory therapy, concomitant steroid therapy, previous biological treatment, and smoking status were not identified as risk factors for experiencing AEs in both infliximab and vedolizumab-treated patients (Table 2). Among infliximab-treated patients, female patients reported adverse events more often than men (*n* = 75, 86% versus *n* = 61, 73.5%, *p* = 0.04, Table 2, Figure 3c). Nevertheless, among vedolizumab-treated patients, gender was not associated with incidence of AE: 34 (82.9%) of the female patients versus 37 (78.7%) of the male patients reported at least one AE (*p* = 0.6, Table 2, Figure 3d). There was no difference between males and females in terms of drug levels in both infliximab and vedolizumab-treated IBD patients (*p* = 0.8 and *p* = 0.1, respectively).

### 3.5. Factors Affecting the Occurrence of AE in the General Cohort

We performed another analysis including both infliximab and vedolizumab-treated patients and examined various clinical and demographic parameters in association with AEs. Among this group, prior biologic therapy and previous IBD-related surgeries were numerically associated with higher occurrence of AEs, although statistical significance was not reached (*p* = 0.08 and *p* = 0.09, respectively, Table S1). In addition, AEs were more common in female patients and in patients with EIM (*p* = 0.01 and *p* < 0.0001, respectively, Table S1). Neither IBD type, disease duration, age, smoking, concomitant immunomodulator therapy, nor concomitant steroid therapy were correlated with AEs (IBD type: *p* = 0.65; disease duration: *p* = 0.3; age: *p* = 0.9; smoking: *p* = 0.29; concomitant immunomodulator therapy: *p* = 0.45; and concomitant steroid therapy: *p* = 0.65, in patients with and without AEs, Table S1).

## 4. Discussion

Biological therapies such as anti-TNFαs and anti-integrins are considered the mainstay of therapy for moderate to severe IBD. Although these drugs have been shown to be highly effective in the induction and maintenance of clinical and endoscopic remission, potential AEs can cause significant morbidity and mortality. Anti-TNFs have been associated with a wide range of AEs, including the increased risk of infections (viral, bacterial, fungal, or opportunistic) [16,17,18]; hematological disorders (leukopenia, thrombocytopenia, and/or anemia as well as non-Hodgkin’s lymphoma) [19,20,21,22,23]; and dermatological AEs including skin malignancies, psoriasis, granuloma annulare, interstitial granulomatous dermatitis, and erythema nodosum [24,25]. Demyelination has been recognized as a neurological AE of anti-TNFs both in the central and peripheral nervous system [26,27]. Decompensated heart failure has also been reported as a possible anti-TNF AE. Moreover, a few case reports described second and third-degree atrioventricular blocks after infliximab therapy [28,29]. Other AEs, such as drug-induced systemic lupus erythematosus, antiphospholipid syndrome, and sarcoidosis, have also been previously described in relation to TNF blockers [30].

Although vedolizumab is a gut selective antibody to α4β7 integrin, previous studies have demonstrated an increased risk of AEs compared to the placebo [3,4]. Vedolizumab treatment has previously been associated with AEs such as infectious colitis and nasophryngitis [31]. A retrospective review of the VICTORY Consortium data reported serious AEs and serious infections in 6% and 4% of vedolizumab-treated patients, respectively. In this study, infectious colitis due to clostridium difficile, Entamoeba histolytica, and Cytomegalovirus were reported. Other AEs including sinusitis, pneumonia, cholangitis, diverticulitis, and transverse myelitis were reported as well [32]. Zeissig and colleagues have demonstrated possible alterations in the innate immunity as a consequence of vedolizumab therapy [33]. These alterations, if proven further on, may be linked with AEs associated with infections and wound-healing.

The current study is a cross-sectional study that examined the association between trough levels of biologics administered consecutively intravenously—namely vedolizumab and infliximab—and possible AEs, as reported by the patients at time of the following infusion. A trend was observed for the association of higher infliximab levels and the occurrence of adverse events (Figure 1a,b). For vedolizumab, levels higher than 18 µg/mL were associated with a higher rate of AEs as well. No specific association was observed between increased drug levels of either infliximab or vedolizumab and specific AEs (neurological symptoms, infectious complications, and musculoskeletal symptoms). Severe AEs documented in the medical records were also analyzed in association with trough levels. Only in a minority of cases, antibiotic administration was indicated to address the reported AE and no cases of hospitalization or change in the treatment regimen were indicated due to AE. Thus, although higher TLs were associated with the occurrence of AEs, those AEs rarely necessitated a change in therapy regimen or any additional clinical measures. However, as severe AEs were a rare outcome (2/169, 1% infliximab; 2/88, 2% vedolizumab), corroborating large-scale studies are required.

To date, only a few studies have examined the possible association between serum drug levels and the risk of developing AEs. In a recent retrospective study by Sengupta and colleagues, vedolizumab serum levels above 15.8 μg/mL were found not associated with the occurrence of AEs, including infections, dermatologic reactions, infusion reactions, malignancy, cardiovascular events, and abnormal liver events [34]. Regarding TNF antagonists, a small number of studies examined the possible relationship between serum drug levels and the risk of developing AEs. A retrospective study by Greener and colleagues also demonstrated no association between increased infliximab levels (above 15 μg/mL) and the risk of AEs. Similar findings were observed in a study by Bodini and colleagues who examined the association between TNF blockers (infliximab and adalimumab) and AEs [8,35]. As these are retrospective studies and not prospective or cross-sectional, recall bias could have affected patients’ reporting.

Patients with known EIM reported more AEs. It should be noted that the questionnaire included questions about symptoms such as joint pain, myalgia, and fatigue (Appendix A), and the above patients may have suffered more from these manifestations as part of their underlying disease. When omitting non-specific and often disease-associated manifestations, namely fatigue and abdominal pain, the association between infliximab levels and the occurrence of adverse events was more pronounced. Similar findings were observed in past studies, in which non-serious AEs, such as fatigue and arthralgia, were frequently reported and not related to drug treatment, but rather were secondary to the underlying disease [5]. AEs were reported more often by females rather than males among infliximab therapy-receiving patients, although no such association was detected among the vedolizumab therapy-receiving group. However, no difference was observed in infliximab and vedolizumab drug levels between males and females. A similar finding was observed in a previous study by Lie and colleagues, which demonstrated that female IBD patients receiving adalimumab reported more AEs and were more likely to discontinue treatment compared to men [36]. In addition, several previous studies suggested female IBD patients had also reported a lower quality of life (as per designated questionnaires), more emotional disturbances linked to their disease, and an increased tendency to fatigue compared to men with IBD [37,38,39]. In addition, among infliximab patients, an association between trough levels below 7 μg/mL and the appearance of a rash/itching was observed. The significance of this finding is not clear and further studies would determine if patients with lower infliximab levels manifested more dermatological manifestations of IBD, such as erythema nodosum or psoriasis, or whether this is an incidental finding.

The present study has several limitations. Firstly, although this is one of the largest cross-sectional studies to date to explore the associations between trough levels of biologics and AEs, the number of patients included in some of the sub-analyses was small, such as the analysis of TL versus significant AEs. Furthermore, the etiology of manifestations such as arthralgia could be the result of the drug or the baseline disease itself. Secondly, a reporting bias may have taken place, with patients over-reporting symptoms when presented with a questionnaire. To overcome this, a sub-analysis for significant adverse events with solid endpoints was performed. Third, given the study design, the risk of long-term complications such as malignancy cannot be discussed. Finally, a selection bias could have also taken place, with patients who were more aware of possible AEs consenting more often to participate. This was minimized by a general explanation of the study’s aims upon enrollment. In addition, only a minority of patients (2.6%) refused to consent.

## 5. Conclusions

This study is the largest cross-sectional study to date that demonstrates that higher drug trough levels of infliximab are associated with the increased rate of AEs. No such association was demonstrated for vedolizumab and no specific AE was found to be correlated with higher levels of either drug. Due to the study design, additional prospective, large-scale, and long-term studies are required, especially with regard to serious AEs including malignancy and opportunistic infections, in order to examine a correlation between drug levels and the accumulative risk of long-term complications.

## Figures and Tables

**Table 1:** Background disposition and clinical characteristics.

Parameter		Infliximab N(%)	Vedolizumab N(%)
Age (years, IQR)		31, 26–41	40, 31–57
Gender (female) ^a^		60 (48%)	36 (51%)
Extraintestinal manifestations ^a^		57 (46%)	33 (46%)
Smoking ^a^		22 (18%)	11 (15%)
Previous surgery ^a^		27 (22%)	17 (24%)
Concomitant immunomodulator therapy ^a^		25 (20%)	5 (7%)
Concomitant methotrexate therapy ^a^		1 (1%)	1 (1%)
Concomitant corticosteroid therapy ^a^		9 (7%)	7 (10%)
Previous anti-TNF therapy ^a^		53 (4%)	46 (65%)
IBD type (UC) ^a^		27 (22%)	37 (52%)
CD—disease location ^b^	Ileal	52 (54%)	15 (44%)
	Colonic	14 (14%)	6 (18%)
	Ileocolonic	30 (31%)	13 (38%)
	Upper GI tract *	1 (1%)	-
CD—disease behavior ^b^	Non-stricturing and non-penetrating	38 (39%)	16 (47%)
	Stricturing	30 (31%)	11 (32%)
	Penetrating	29 (30%)	7 (21%)
UC—disease location ^c^	Extensive colitis	17 (63%)	21 (57%)
	Left-sided colitis	10 (37%)	15 (41%)
	Proctitis	0 (0%)	1 (3%)
Disease duration (years, IQR)		8, 4–15	9, 4–18
Median trough level, µg/mL (IQR)		5.3 μg/mL (3.1–9.1)	47.9 μg/mL (28.2–87.2)

Abbreviations: IQR = interquartile range; IBD = inflammatory bowel disease; UC = ulcerative colitis; CD = Crohn disease; and GI = gastrointestinal. * Upper GI tract solely. Infliximab percentage noted is out of: ^a^ 124 IBD patients; ^b^ 97 CD patients; and ^c^ 27 UC patients. Vedolizumab percentage noted is out of: ^a^ 71 IBD patients; ^b^ 34 CD patients; and ^c^ 37 UC patients.

**Table 2:** Association of baseline parameters with adverse events.

Parameter		Infliximab				Vedolizumab			
		With AE N(%)/median	Without AE N(%)/median	OR (CI)/IQR	*p*-value	With AE N(%)/median	Without AE N(%)/median	OR (CI)/IQR	*p*-value
Age		30	33	25–41; 28–41	0.4	47	38	31–61; 51–46	0.3
Disease duration		8	6	5–15; 4–16	0.7	9	6	5–18; 3–16	0.4
Gender	Female	75/86 (87.2%)	11/86 (12.7%)	2.2 (1.0–4.9)	**0.04**	34/41 (82.9%)	7/41 (17.1%)	1.3 (0.4–3.8)	0.6
	Male	61/83 (73.5%)	22/83 (26.5%)			37/47 (78.7%)	10/47 (21.3%)		
Extraintestinal manifestations	Yes	71/77 (92.2%)	6/77 (7.8%)	5.1 (2.0–13.3)	**0.0006**	37/39 (94.9%)	2/39 (5.1%)	8.1 (1.7–38.3)	**0.008**
	No	64/92 (69.6%)	28/92 (30.4%)			34/49 (69.4%)	15/49 (30.6%)		
Smoking	Yes	24/29 (82.8%)	5/29 (17.2%)	1.2 (0.4–3.5)	0.7	13/14 (92.9%)	1/14 (7.1%)	0.2 (0.0–2.3)	0.2
	No	111/140 (79.3%)	29/140 (20.7%)			58/74 (78.4%)	16/74 (21.6%)		
Previous surgery	Yes	32/37 (86.5%)	5/37 (13.5%)	1.8 (0.6–5.0)	0.3	19/21 (90.5%)	2/21 (9.5%)	2.7 (0.5–13.1)	0.2
	No	103/132 (78%)	29/132 (22%)			52/67 (77.6%)	15/67 (22.4%)		
Concomitant immunomodulator therapy (Methotrexate/Azathioprine/purinethol)	Yes	26/36 (72.2%)	10/36 (27.8%)	0.6 (0.2–1.3)	0.2	9/10 (90%)	1/10 (10%)	2.3 (0.2–19.7)	0.4
	No	109/133 (82%)	24/133 (18%)			62/78 (79.5%)	16/78 (20.5%)		
Concomitant corticosteroid therapy	Yes	9/11 (81.8%)	2/11 (18.2%)	1.1 (0.2–5.5)	0.9	7/8 (87.5%)	1/8 (12.5%)	1.7 (0.2–15.2)	0.6
	No	126/158 (79.7%)	32/158 (20.3%)			64/80 (80%)	16/80 (20%)		
Previous anti-TNF therapy	Yes	61/72 (84.7%)	11/72 (15.3%)	1.7 (0.7–3.8)	0.2	48/57 (84.2%)	9/57 (15.8%)	1.8 (0.6–5.4)	0.3
	No	74/97 (76.3%)	23/97 (23.7%)			23/31 (74.2%)	8/31 (25.8%		
IBD type	CD	109/137 (79.6%)	28/137 (20.4%)	1.1 (0.4–2.9)	0.8	35/41 (85.4%)	6/41 (14.6%)	0.5 (0.1–1.6)	0.3
	UC	26/32 (81.2%)	6/32 (18.8%)			36/47 (76.6%)	11/47 (23.4%)		

Abbreviations: AE = adverse events; TNF = Tumor Necrosis Factor alpha; IBD = inflammatory bowel disease; CD = Crohn’s disease; UC = ulcerative colitis; OR = odds ratio; and IQR = interquartile range. The bold is intended to emphesis statistically significant results (*p*-value below 0.05).

## Supplementary Materials

The following are available online at https://www.mdpi.com/article/10.3390/jcm10184265/s1, Table S1: Parameters of infliximab and vedolizumab-treated patients and the association of reporting adverse events.

## Appendix A

An English translation of the questionnaire filled out by IBD patients under treatment of infliximab or vedolizumab.

**INFLIXIMAB** **/** **VEDOLI** **Z** **UMAB LEVELS QUESTIONNAIRE**

I.D. No.:_______________ Date:____________________

Dear patient,

We are conducting a questionnaire aimed at examining a possible link between possible symptoms and serum drug levels of the biological drugs administered. Drug levels measured in your serum today will be collected and analyzed concurrently with the information provided by you in this questionnaire.

The information provided will be kept anonymous to protect your privacy.

In answering this questionnaire, you give your consent to the use of the information provided (by you) in the questionnaire and serum drug levels, as above.

In the questionnaire there is a list of symptoms/possible adverse events, in your reply, please refer to the period of the last two months.

**Adverse event/symptom**	**Did you experience: yes/no**	**Did it cause/resulted in treatment cessation: yes/no**	**Did you receive treatment for this adverse event, if yes, please elaborate:**
headache			
dizziness			
abdominal pain			
nausea or vomiting			
fever			
cough			
dyspnea			
sinusitis			
pneumonia			
pharyngitis			
blushing			
increased fatigue			
rash/pruritis			
urinary tract infection			
arthralgia			
arthritis			
peripheral edema			
parasthesia			
bone pain			
myalgia			
muscle weakness			
Other			
